# Supplementary material for: Divergent folding-mediated epistasis among unstable membrane protein variants
Source: eLife. 2024 Jul 30;12:RP92406. doi: 10.7554/eLife.92406 (PMC11288631; doi:10.7554/eLife.92406)
Supplement: Supplementary file 1. [file elife-92406-supp1.docx]

**Supplementary File 1. Predicted and measured apparent transfer free energies of mGnRHR TM6.**

| **Insert TMD** | **K_app_** ‡  **(Int. _G1_ / Int. _G2_)** | **ΔG_app_** ‡  **(kcal/ mol)** | **ΔG_app, pred_** †  **(kcal/ mol)** |
| --- | --- | --- | --- |
| WT TMD6 | 1.2 ± 0.2 | -0.1 ± 0.1 | 0.26 |
| V276T TMD6 | 0.7 ± 0.1 | 0.2 ± 0.1 | 0.805 |

‡Values represent the average of three experimental replicates, and errors represent the standard deviation.

†Values predicted from the TM6 sequence using the ΔG Predictor.
