# Supplementary material for: Divergent folding-mediated epistasis among unstable membrane protein variants
Source: eLife. 2024 Jul 30;12:RP92406. doi: 10.7554/eLife.92406 (PMC11288631; doi:10.7554/eLife.92406)
Supplement: Supplementary file 2. [file elife-92406-supp2.docx]

**Supplementary File 2. Solvent accessible surface area of mGnRHR residues.**

| **Position** | **Residue** | **Percent Relative Accessibility (REL)** |
| --- | --- | --- |
| 13 | His | 91.1 |
| 14 | Cys | 63.9 |
| 15 | Ser | 21.9 |
| 16 | Ala | 32.1 |
| 17 | Ile | 67.3 |
| 18 | Asn | 76.5 |
| 19 | Asn | 1.3 |
| 20 | Ser | 15.6 |
| 21 | Ile | 3.9 |
| 22 | Pro | 27.2 |
| 23 | Leu | 3.2 |
| 24 | Ile | 50.7 |
| 25 | Gln | 33.3 |
| 26 | Gly | 9.0 |
| 27 | Lys | 69.8 |
| 28 | Leu | 7.6 |
| 29 | Pro | 44.6 |
| 30 | Thr | 45.0 |
| 31 | Leu | 13.5 |
| 32 | Thr | 84.7 |
| 33 | Val | 69.0 |
| 34 | Ser | 13.8 |
| 35 | Gly | 4.5 |
| 36 | Lys | 80.6 |
| 37 | Ile | 51.7 |
| 38 | Arg | 7.0 |
| 39 | Val | 29.3 |
| 40 | Thr | 49.0 |
| 41 | Val | 31.7 |
| 42 | Thr | 12.3 |
| 43 | Phe | 59.6 |
| 44 | Phe | 62.2 |
| 45 | Leu | 3.5 |
| 46 | Phe | 27.5 |
| 47 | Leu | 64.6 |
| 48 | Leu | 31.0 |
| 49 | Ser | 0.0 |
| 50 | Thr | 31.0 |
| 51 | Ala | 51.6 |
| 52 | Phe | 29.7 |
| 53 | Asn | 0.0 |
| 54 | Ala | 30.6 |
| 55 | Ser | 40.6 |
| 56 | Phe | 7.0 |
| 57 | Leu | 11.6 |
| 58 | Leu | 57.0 |
| 59 | Lys | 54.6 |
| 60 | Leu | 0.7 |
| 61 | Gln | 44.7 |
| 62 | Lys | 72.6 |
| 63 | Trp | 26.1 |
| 64 | Thr | 49.3 |
| 65 | Gln | 81.5 |
| 66 | Lys | 53.8 |
| 67 | Arg | 91.8 |
| 68 | Lys | 59.2 |
| 69 | Lys | 34.7 |
| 70 | Gly | 24.7 |
| 71 | Lys | 71.5 |
| 72 | Lys | 42.9 |
| 73 | Leu | 3.7 |
| 74 | Ser | 10.8 |
| 75 | Arg | 18.0 |
| 76 | Met | 4.0 |
| 77 | Lys | 7.5 |
| 78 | Val | 9.2 |
| 79 | Leu | 0.1 |
| 80 | Leu | 2.2 |
| 81 | Lys | 21.5 |
| 82 | His | 14.5 |
| 83 | Leu | 1.5 |
| 84 | Thr | 0.0 |
| 85 | Leu | 33.8 |
| 86 | Ala | 0.2 |
| 87 | Asn | 6.0 |
| 88 | Leu | 17.4 |
| 89 | Leu | 37.1 |
| 90 | Glu | 4.4 |
| 91 | Thr | 0.1 |
| 92 | Leu | 52.1 |
| 93 | Ile | 44.3 |
| 94 | Val | 3.0 |
| 95 | Met | 3.0 |
| 96 | Pro | 49.0 |
| 97 | Leu | 16.9 |
| 98 | Asp | 3.0 |
| 99 | Gly | 16.4 |
| 100 | Met | 27.2 |
| 101 | Trp | 14.8 |
| 102 | Asn | 5.0 |
| 103 | Ile | 21.9 |
| 104 | Thr | 43.7 |
| 105 | Val | 5.6 |
| 106 | Gln | 12.7 |
| 107 | Trp | 0.5 |
| 108 | Tyr | 57.0 |
| 109 | Ala | 17.3 |
| 110 | Gly | 49.6 |
| 111 | Glu | 36.5 |
| 112 | Phe | 69.9 |
| 113 | Leu | 34.1 |
| 114 | Cys | 0.2 |
| 115 | Lys | 21.2 |
| 116 | Val | 11.2 |
| 117 | Leu | 9.3 |
| 118 | Ser | 14.0 |
| 119 | Tyr | 24.0 |
| 120 | Leu | 30.6 |
| 121 | Lys | 3.3 |
| 122 | Leu | 5.6 |
| 123 | Phe | 23.2 |
| 124 | Ser | 6.1 |
| 125 | Met | 6.5 |
| 126 | Tyr | 0.0 |
| 127 | Ala | 0.0 |
| 128 | Pro | 7.3 |
| 129 | Ala | 1.3 |
| 130 | Phe | 21.6 |
| 131 | Met | 0.3 |
| 132 | Met | 5.8 |
| 133 | Val | 11.5 |
| 134 | Val | 6.7 |
| 135 | Ile | 2.4 |
| 136 | Ser | 0.0 |
| 137 | Leu | 43.5 |
| 138 | Asp | 2.1 |
| 139 | Arg | 9.4 |
| 140 | Ser | 22.0 |
| 141 | Leu | 21.4 |
| 142 | Ala | 15.6 |
| 143 | Ile | 14.6 |
| 144 | Thr | 31.0 |
| 145 | Gln | 59.5 |
| 146 | Pro | 50.1 |
| 147 | Leu | 95.7 |
| 148 | Ala | 35.3 |
| 149 | Val | 67.0 |
| 150 | Gln | 65.6 |
| 151 | Ser | 4.0 |
| 152 | Asn | 41.5 |
| 153 | Ser | 40.8 |
| 154 | Lys | 85.6 |
| 155 | Leu | 40.3 |
| 156 | Glu | 2.4 |
| 157 | Glm | 40.2 |
| 158 | Ser | 41.0 |
| 159 | Met | 33.0 |
| 160 | Ile | 0.8 |
| 161 | Ser | 45.5 |
| 162 | Leu | 46.4 |
| 163 | Ala | 1.5 |
| 164 | Trp | 17.8 |
| 165 | Ile | 52.0 |
| 166 | Leu | 37.9 |
| 167 | Ser | 0.0 |
| 168 | Ile | 60.8 |
| 169 | Val | 58.1 |
| 170 | Phe | 24.9 |
| 171 | Ala | 4.4 |
| 172 | Gly | 45.4 |
| 173 | Pro | 23.8 |
| 174 | Gln | 3.7 |
| 175 | Leu | 40.7 |
| 176 | Tyr | 29.5 |
| 177 | Ile | 0.0 |
| 178 | Phe | 13.1 |
| 179 | Arg | 36.4 |
| 180 | Met | 21.9 |
| 181 | Ile | 7.9 |
| 182 | Tyr | 64.6 |
| 183 | Leu | 40.2 |
| 184 | Ala | 0.3 |
| 185 | Asp | 20.1 |
| 186 | Gly | 97.0 |
| 187 | Ser | 26.6 |
| 188 | Gly | 2.2 |
| 189 | Pro | 80.5 |
| 190 | Thr | 70.8 |
| 191 | Val | 63.0 |
| 192 | Phe | 11.8 |
| 193 | Ser | 2.2 |
| 194 | Gln | 5.3 |
| 195 | Cys | 9.0 |
| 196 | Val | 6.3 |
| 197 | Thr | 0.5 |
| 198 | His | 4.9 |
| 199 | Cys | 0.0 |
| 200 | Ser | 21.3 |
| 201 | Phe | 54.6 |
| 202 | Pro | 38.7 |
| 203 | Gln | 40.4 |
| 204 | Trp | 43.1 |
| 205 | Trp | 43.3 |
| 206 | His | 19.3 |
| 207 | Gln | 0.0 |
| 208 | Ala | 3.9 |
| 209 | Phe | 31.8 |
| 210 | Tyr | 26.2 |
| 211 | Asn | 0.7 |
| 212 | Phe | 57.2 |
| 213 | Phe | 46.1 |
| 214 | Thr | 5.6 |
| 215 | Phe | 19.7 |
| 216 | Gly | 19.0 |
| 217 | Cys | 24.8 |
| 218 | Leu | 4.1 |
| 219 | Phe | 6.3 |
| 220 | Ile | 34.9 |
| 221 | Ile | 50.2 |
| 222 | Pro | 7.7 |
| 223 | Leu | 20.8 |
| 224 | Leu | 52.4 |
| 225 | Ile | 36.8 |
| 226 | Met | 2.7 |
| 227 | Leu | 55.4 |
| 228 | Ile | 58.2 |
| 229 | Cys | 14.9 |
| 230 | Asn | 7.5 |
| 231 | Ala | 35.5 |
| 232 | Lys | 63.7 |
| 233 | Ile | 0.1 |
| 234 | Ile | 34.0 |
| 235 | Phe | 70.0 |
| 236 | Ala | 25.7 |
| 237 | Leu | 20.4 |
| 238 | Thr | 51.5 |
| 239 | Arg | 73.4 |
| 240 | Val | 42.9 |
| 241 | Leu | 74.1 |
| 242 | His | 96.1 |
| 243 | Gln | 64.9 |
| 244 | Asp | 23.5 |
| 245 | Pro | 44.3 |
| 246 | Arg | 67.7 |
| 247 | Lys | 51.7 |
| 248 | Leu | 41.1 |
| 249 | Gln | 46.3 |
| 250 | Leu | 51.1 |
| 251 | Asn | 26.2 |
| 252 | Gln | 76.8 |
| 253 | Ser | 71.5 |
| 254 | Lys | 61.6 |
| 255 | Asn | 93.2 |
| 256 | Asn | 43.8 |
| 257 | Ile | 55.2 |
| 258 | Pro | 12.6 |
| 259 | Arg | 72.2 |
| 260 | Ala | 39.4 |
| 261 | Arg | 29.7 |
| 262 | Leu | 31.1 |
| 263 | Arg | 8.3 |
| 264 | Thr | 13.3 |
| 265 | Leu | 4.2 |
| 266 | Lys | 55.6 |
| 267 | Met | 2.4 |
| 268 | Thr | 5.2 |
| 269 | Val | 39.8 |
| 270 | Ala | 39.5 |
| 271 | Phe | 3.3 |
| 272 | Ala | 13.6 |
| 273 | Thr | 51.0 |
| 274 | Ser | 9.2 |
| 275 | Phe | 6.0 |
| 276 | Val | 28.4 |
| 277 | Val | 57.2 |
| 278 | Cys | 1.4 |
| 279 | Trp | 2.4 |
| 280 | Thr | 17.6 |
| 281 | Pro | 20.2 |
| 282 | Tyr | 14.6 |
| 283 | Tyr | 6.5 |
| 284 | Val | 29.8 |
| 285 | Leu | 3.1 |
| 286 | Gly | 7.2 |
| 287 | Ile | 26.7 |
| 288 | Trp | 56.9 |
| 289 | Tyr | 18.0 |
| 290 | Trp | 23.1 |
| 291 | Phe | 35.9 |
| 292 | Asp | 51.7 |
| 293 | Pro | 14.0 |
| 294 | Glu | 81.8 |
| 295 | Met | 41.9 |
| 296 | Leu | 11.8 |
| 297 | Asn | 53.7 |
| 298 | Arg | 48.4 |
| 299 | Val | 65.5 |
| 300 | Ser | 16.8 |
| 301 | Glu | 3.0 |
| 302 | Pro | 17.3 |
| 303 | Val | 34.4 |
| 304 | Asn | 4.1 |
| 305 | His | 2.9 |
| 306 | Phe | 48.8 |
| 307 | Phe | 24.6 |
| 308 | Phe | 6.8 |
| 309 | Leu | 11.4 |
| 310 | Phe | 36.9 |
| 311 | Ala | 0.1 |
| 312 | Phe | 1.9 |
| 313 | Leu | 28.8 |
| 314 | Asn | 1.3 |
| 315 | Pro | 1.2 |
| 316 | Cys | 2.2 |
| 317 | Phe | 36.7 |
| 318 | Asp | 1.2 |
| 319 | Pro | 4.4 |
| 320 | Leu | 52.2 |
| 321 | Ile | 18.7 |
| 322 | Tyr | 0.8 |
| 323 | Gly | 31.6 |
| 324 | Tyr | 66.1 |
| 325 | Phe | 26.3 |
| 326 | Ser | 62.2 |
| 327 | Leu | 89.8 |
